# Plant–Earthworm Interaction Favors Invasive Alien Plants Over Natives in Cd‐Contaminated Environments

**DOI:** 10.1002/ece3.71538

**Published:** 2025-06-30

**Authors:** Rui‐Feng Zhang, Ai‐Di‐Na Yisilamu, Cong‐Ying Zhao, Yu‐Jian Guo, Xue Zhang, Shou‐Shuai Zhang, Zhao‐Gui Yan, Yan‐Feng Bai, Yong‐Jian Wang

**Affiliations:** ^1^ College of Horticulture and Forestry Sciences Huazhong Agricultural University Wuhan China; ^2^ Wuhan Tortoise Mountain Scenic Area Administrate Office Wuhan China; ^3^ Research Institute of Forestry Chinese Academy of Forestry Beijing China

**Keywords:** adaptive strategy, earthworm, functional group, heavy metal, invasive species, plant growth

## Abstract

Plant–earthworm interaction confers fitness advantages to the plants, including growth promotion, nutrient uptake, tolerance, and resistance to heavy metal (HM) contamination. Plant dominance and plant–soil nutrient cycle processes of plant invasion in contaminated environments can be mediated by plant–earthworm interaction. However, little is known about whether different functional groups of invasive plants can gain an advantage in HM‐contaminated soils when regulated by plant–earthworm interaction. We conducted an experiment to examine the effects of Cadmium (Cd) (with or without) and earthworms (with or without) on the growth of both native and invasive species of legume, grass, and forb functional groups. We found that Cd reduced the biomass of native species and changed the root mass fraction and root‐shoot ratio, while earthworms increased the aboveground mass of invasive species. When contaminated with Cd, earthworms increased the aboveground mass of invasive nonlegumes (grasses and forbs) while reducing the biomass loss of invasive legumes. In turn, invasive plants had a positive effect on earthworm mass. The mutually beneficial relationship between earthworms and invasive species in Cd‐contaminated environments can enhance the competitive edge of invasive species over natives. This may facilitate invasive species spread, potentially risking Cd contamination and food chain transmission, threatening ecosystems biodiversity, environmental health, and human health. Thus, earthworm management may be an important measure to control the spread of invasive species in Cd‐contaminated areas, particularly invasive nonlegumes.

## Graphical abstract

We investigated the impact of Cd pollution and earthworms on the growth of native and invasive plant species across different functional groups. Our findings revealed a mutually beneficial relationship between earthworms and invasive species in Cd‐contaminated environments, which enhances the competitive advantage of invasives over natives. These results offer novel perspectives on managing invasive plants in areas with heavy metal pollution, underscoring the critical connection between metal contamination and the global spread of invasive species.

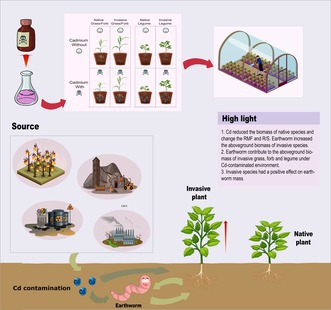

## 1 Introduction

Heavy metal (HM) contamination poses a serious threat to global biodiversity and human health (Xie et al. 2016; Siddig et al. 2025). Cadmium (Cd) is considered the most harmful among many HMs due to its high toxicity and bioavailability (Ali et al. 2015). The input of Cd into soil and water comes primarily from combustion discharge, sewage sludge, landfill, and mining (Gupta and Nayak 2012; Kubier et al. 2019; Zeng et al. 2021; Shi et al. 2022). Although not involved in biological processes, Cd is widespread in ecosystems and is toxic to plants even at low concentrations (Rasafi et al. 2022). Cd can accumulate and be absorbed into plants via soil. Once in the plant, Cd hinders the uptake of nutrients, affects plant growth, and induces symptoms of poisoning (Qin et al. 2020). Specifically, Cd can reduce the growth and developmental rate by altering the morphological structure of the root, decreasing the photosynthetic rate, and modifying the plant growth strategy by altering the root‐shoot (R/S) ratio (Gallego et al. 1996; Ji et al. 2017; Zhang et al. 2020). The extent of the effects depends on the characteristics of the plant and is regulated by the external environment (Tran and Popova 2013). Furthermore, Cd can be transferred through the food chain, leading to chronic toxic effects on animals (Redondo‐Gomez et al. 2010).

Earthworms are common in soil ecosystems and have positive effects on soil structure, nutrient cycling, and plant growth (Medina‐Sauza et al. 2019; Jacquiod et al. 2020). Studies have demonstrated that earthworms can accelerate the rate of organic matter decomposition and increase mineralization (Chen et al. 2023). Their activities, such as burrowing, excreting, and foraging, can alter soil water conditions and resource availability, especially in the carbon and nitrogen cycle (Zhang et al. 2013; Rodriguez et al. 2020). The metabolites, such as urine, feces, and mucus, which contain a high concentration of nitrogenous substances like NH^4+^ and urea, increase the available nutrients in the soil, indirectly benefiting plant growth (Wurst et al. 2004; Blouin et al. 2013; Zhang et al. 2013). Furthermore, as “ecosystem engineers”, earthworms are utilized to address HM contamination (Butt et al. 1995; Sharma et al. 2017; Wu et al. 2020). Earthworm activities and metabolism mitigate HM toxicity in soil ecosystems by altering pH and enhancing organic matter degradation, thereby decreasing metal mobility and bioavailability, which indirectly benefits plant growth under HM stress (Sizmur et al. 2011; Wang et al. 2023). Additionally, earthworms can also enhance the absorption and degradation of HMs by promoting plant growth and increasing the population of microorganisms (Jusselme et al. 2012). However, HMs are toxic to earthworms, inhibiting their growth and even causing mortality (Wang et al. 2018). The interaction between HMs and earthworms, where earthworms alleviate HM contamination while being susceptible to its toxicity, suggests the complex role of them in soil ecosystems and plant performance (Lemtiri et al. 2016).

Plant invasion is considered a significant ecological problem and one of the leading threats to biodiversity worldwide (Powell et al. 2011). The invasion process is influenced by environmental and biological factors (Wang et al. 2019). For example, studies have found that nutrient deficiency and natural enemies may promote the invasion success of exotic species in native communities (Xu et al. 2024), and HM pollution and interspecific competition can enhance the competitive advantage of invasive plants (Wang et al. 2021). Due to their high resource access and accelerated growth rates, invasive species often establish rapidly in non‐native environments with abundant resources (van Boheemen et al. 2019). Similarly, invasive plants are generally believed to possess a competitive advantage in resource‐limited or stressed environments compared with native species (Piola and Johnston 2008; Prabakaran et al. 2019; Zhang et al. 2024). In general, certain invasive plants are believed to have a high tolerance of HMs, and due to their high adaptability and well‐developed root systems, they minimize damage by enhancing interactions with microorganisms and improving nutrient absorption to promote growth (Han et al. 2021; Wang et al. 2021; Wang, Yang, et al. 2022; Wang, Yu, et al. 2022; Afzal et al. 2023). Furthermore, some invasive species are immune to toxic effects by collecting large amounts of HMs in aerial organs (Khan et al. 2023). These strategies generally make invasive plants more resilient and tolerant to HM stress (Li et al. 2022; Rasafi et al. 2022).

The performances of plants in HM contaminated areas are closely related to characteristics and vary significantly between different functional groups (Al‐Lami et al. 2021). Research indicates that under HM contamination, the presence of forbs leads to a reduction in the biomass of communities, while grasses and legumes mixed communities exhibit higher biomass (Frérot et al. 2006). Furthermore, certain species of grasses, such as *Holcus lanatus* and *Vetiveria zizanioides* , demonstrate exceptional tolerance to HMs, particularly Cd (Danh et al. 2009; Rahman and Khan 2023). These divergent responses among species may reshape vegetation dynamics and competitive patterns in contaminated areas, potentially influencing biodiversity and ecosystem stability. However, the differences in response mechanisms among different functional groups under HM stress remain poorly understood.

Here, we conducted a study that assessed the effects of Cd and earthworm on the growth and development of six invasive‐native species pairs belonging to different functional groups (grass, legume, and forb) in a greenhouse. We evaluated the differential effects of Cd and earthworms on native and invasive plants by measuring biomass and calculating the R/S, root mass fraction (RMF) and tolerance indices. The invasive species we selected are globally distributed and have had adverse ecological impacts in China (Wan et al. 2012; Ma 2013). The selected earthworms ( *Pheretima guillelmi* ) are native to China and are commonly used to assess soil HM and contamination control (Zheng and Li 2009). We hypothesized that (i) earthworms enhance plant tolerance to Cd, and invasive species may benefit more from earthworms compared with native species; (ii) invasive grass demonstrates greater tolerance to Cd than the other two functional groups; and (iii) the response of legumes may be different from nonlegumes due to nitrogen mineralization caused by earthworms.

## 2 Materials and Methods

### 2.1 Species Selection and Culture

Twelve species were selected, comprising six pairs of native and invasive species (Table S1). Every pair contains a native and an invasive species of the same family. All selected species have a wide distribution in the wild and co‐occur in natural habitats. The invasiveness of each species was based on the Flora of China (www.efloras.org) and the Invasive Alien Species of China (IASC) (www.iplant.cn/ias) (Wan et al. 2012; Ma 2013). All selected species belong to three functional groups: grass, legume, and forb, with each functional group comprising two pairs of invasive‐native cogeneric or confamilial species (Table S1). The selection of closely related species partially reduces the impact of interspecific differences on experimental outcomes. All plant materials were collected in the fields or purchased from commercial seed companies.

We propagated these species by clonal propagation and sexual reproduction aligning with their natural reproductive strategies (Table S1). For those species with clonal reproduction, we cut the stolons and rhizomes of the original plant clones into single‐node fragments. Fifty healthy single‐node fragments per species were planted in plastic trays filled with peat soil. For other species with sexual reproduction, we sowed about 50 seeds per species in each plastic tray. All trays were placed in a climate chamber at Huazhong Agricultural University in Wuhan, Hubei Province, China. The temperature of the climate chamber was set between 22°C and 28°C. After three weeks of growth, 16 seedlings with three leaves and of similar size were selected from each species. In total, 192 seedlings or ramets were randomly selected and assigned to different treatments.

### 2.2 Cadmium Heavy Metal

We used round pots (21 cm in diameter and 16 cm in depth) to plant species. Each pot was filled with 3.0 L of field soil and sand (1:2, v/v). Before the experiment, HMs were measured in the planting soil, and the concentration of Cd or other HMs was not detected. According to the “Soil Contamination Risk Control Standards for Soil Environmental Quality Construction Land” (GB 36600–2018 in China, and the concentration is 20–172 mg/kg), we set the added concentration of Cd at 80 mg/kg. While the selected concentration was at a higher level, it was within the range commonly used in similar experiments (Kubier et al. 2019; Wang et al. 2021). The Cd solution was made by CdCl_2_·2.5H_2_O AR and DI Water, fully stirred, and added to the pots. At the same time, the pots of the control group were filled with the same amount of DI water. Following Cd addition, all pots underwent a 15‐day aging period prior to seedling transplantation to reduce Cd mobility and acute toxicity, better simulating natural contamination conditions.

### 2.3 Earthworm

We selected the endogeic earthworm *P. guillelmi* as experimental materials because they are widely distributed in China and are known to have superior transportation and decomposition ability (Ma et al. 2002; Zheng and Li 2009). The earthworms were bought from Runlong Ecological Agriculture Co. Ltd., Jiangsu province, China. The earthworms were cultured in the original soil in an incubator at a temperature of 20°C ± 1°C until the start of the experiment. Healthy earthworms of similar size were randomly selected and assigned to the treatment. The initial biomass of each earthworm was measured. The mean initial biomass of the earthworms was 0.42 ± 0.032 g.

### 2.4 Experimental Design

The experiment was carried out in the greenhouse of the Department of Horticulture of Huazhong Agricultural University. The greenhouse maintained temperatures between 22°C and 28°C, with 50 klx daylight of 12 h and relative humidity ranging from 35.4% to 55.8%. After the Cd aging period, the seedlings were transferred to pots and allowed to recover for 10 days. Then an earthworm was added to each pot. A total of 192 pots were set up with the following treatments: 2 origins (invasive vs. native) × 2 pairs of plant species pairs × 3 functional groups (grass, legume, and forb) × 2 Cd addition treatments (with vs. without) × 2 earthworm addition treatments (with vs. without) × 4 replicates. To prevent the loss of Cd in the soil during the experiment, the daily watering amount was adjusted to 80% of the field capacity. Gauze covered the bottom of the pots to prevent the earthworms from escaping. To minimize the impact of uneven light on plant growth, the positions of the pots were periodically rotated.

The experiment lasted for 12 weeks, and all materials were harvested at the end of the experiment. At harvest, the aboveground mass and the belowground mass of the plants were sorted and measured separately. All materials were dried at 80°C for 72 h and weighed. The earthworms were carefully removed from the soil, gently washed, weighed for fresh weight, and calculated the change of mass. The R/S ratio, RMF, and tolerance index (plants and earthworms) were also calculated as follows:

R/Sratio=Belowground mass/Aboveground mass

RMF=Belowground mass/Total biomass

$$\text{Tolerance index of plants}={\text{Total biomass}}_{\text{with}\;\mathrm{Cd}}/{\text{Total biomass}}_{\text{without}\;\mathrm{Cd}}$$

$$\text{Tolerance index of earthworms}=(\text{The change}\;{\text{of mass)}}_{\text{with}\;\mathrm{Cd}}/(\text{The change}\;{\text{of mass)}}_{\text{without}\;\mathrm{Cd}}$$

For plants, a higher tolerance index reflected superior growth performance under Cd stress. For earthworms, due to differences in initial masses, final biomass could not serve as a reliable measure of Cd tolerance. We therefore quantified earthworm tolerance using mass change (initial mass minus final mass). As all earthworms exhibited mass reduction by experiment termination, a higher tolerance index indicated greater mass loss (representing reduced Cd tolerance).

### 2.5 Statistical Analysis

We performed the statistical analysis in R 4.0.2. To test for differences in growth performance between native and alien invasive plant species in each functional group, we fitted linear mixed effect models using the nlme package (Pinheiro et al. 2021). The response variables in the model were aboveground biomass, belowground biomass, total biomass of plants, RMF, R/S, the lost mass of earthworms, and tolerance indices (plants and earthworms). In order to satisfy the normal distribution hypothesis, all variables of plants were squared‐root transformed, while all tolerance indices and the lost mass of earthworms were logarithmically transformed. The origin of the species (invasive and native), the addition of Cd, the addition of earthworms, and the functional groups and their interactions were considered as fixed effects in the plant biomass models. We categorized species as either legume or nonlegume for Functional group 1 (F1) and as grass or forb for Functional group 2 (F2) to test our hypothesis. To account for the phylogenetic nonindependence among plant species and the nonindependence of replicates within the same species, species nested within genus were incorporated as random factors in all models. To minimize the effect of the position of the pot on the analyses, the pot number was included as a random effect. To meet the homoskedasticity assumption, we included variance structures modeling different variances per species in all models using the “varIdent” function (Pinheiro et al. 2021). In the earthworm mass model, we excluded the treatment without earthworms and removed the fixed effect of “earthworm addition” while keeping the remaining factors unchanged. We evaluated fixed effects and interactions using likelihood ratio tests in all linear mixed models, selected models via AIC minimization to balance complexity and fit, and adopted *p* < 0.05 as the significance threshold.

### 2.6 Structural Equation Model

To investigate the relationships among variables of all treatments in invasive and native species, we performed piecewise structural equation models (SEMs) in the piecewise SEM package (Lefcheck 2016). Prior to the analysis, we established the conceptual SEM as the foundation (Figure S1). To examine the impacts of Cd and earthworms and their interaction on plant biomass, we hypothesized distinct pathways that lead to root mass, RMF, R/S, and total mass. We posited that Cd and earthworms influence total biomass both directly (via toxicity) and indirectly (by altering aboveground and belowground allocation), while accounting for potential feedback between plant and earthworm biomass. To elucidate the differences between native and invasive species under different treatments, we used four SEMs to analyze plant data from different origins using species as a random factor. The differences between different functional groups were analyzed in similar ways. When fitting the models, we initially calculated the correlations between variables and eliminated the variables exhibiting collinearity, while improving the initial hypothesis model. The model fits of the final candidate were assessed using directed separation tests, Fisher's C tests, and AIC comparison. The best performing model was selected based on the actual reasonable relationship between the variables and the AIC. Finally, we completed the optimization of the models by removing any paths that had no significant effect on the AIC (Fahey et al. 2020).

## 3 Results

**FIGURE 1:**
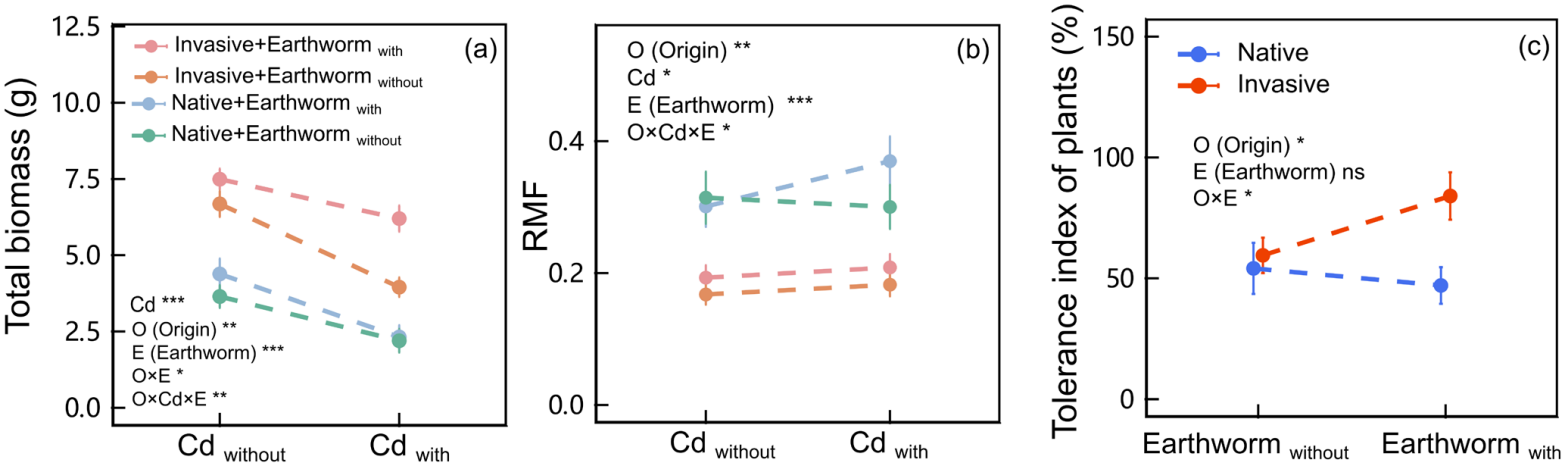
Total biomass (panel a) and root mass fraction (RMF) (panel b) of all invasive and native plant species under earthworm addition (with vs. without) and Cd contamination (with vs. without) conditions. The points of different colors represented combinations of plant origin (native vs. invasive) and earthworm addition, and the horizontal coordinate represented the levels of Cd contamination. The tolerance index to Cd of plants between native and invasive species under earthworm addition or not (panel c). Values were means ± standard error (SE). *p < 0.05, **p < 0.01, ***p < 0.001, ns *p* > 0.1.

Overall, alien invasive plants showed significantly higher biomass but lower RMF and R/S compared to native plants (*χ* ^*2*^ = 11.982, *p* < 0.01and *χ* ^*2*^ = 10.709, *p* < 0.01 in Table 1; *χ* ^*2*^ = 10.776, *p* < 0.01 in Table S2; Figure 1a; Figure S2). Earthworm presence increased and Cd contamination decreased plant biomass (*χ* ^*2*^ = 20.276, *p* < 0.001and *χ* ^*2*^ = 78.068, *p* < 0.001 in Table 1; Figure 1a).

**TABLE 1:** Effects of species origin (invasive vs. native), Cd contamination (with vs. without), earthworm addition (with vs. without), plant functional group1 (F1: legume vs. nonlegume), plant functional group2 (F2: forb vs. grass) and their interactions on total biomass, aboveground biomass, and root mass fraction (RMF) of plant species.

Fixed effects	Total biomass		Aboveground biomass		RMF	
	*χ* ^*2*^	*p*	*χ* ^*2*^	*p*	*χ* ^*2*^	*p*
Species origin (O)	**11.982**	**0.001**	**18.915**	**0.000**	**10.799**	**0.001**
Cd contamination (Cd)	**78.068**	**0.000**	**71.403**	**0.000**	**6.194**	**0.013**
Functional group1 (F1)	**5.765**	**0.016**	1.163	0.281	**4.229**	**0.040**
Functional group2 (F2)	0.122	0.727	0.244	0.621	0.406	0.524
Earthworm (E)	**20.276**	**0.000**	**16.925**	**0.000**	**3.937**	**0.047**
O × Cd	1.494	0.222	1.419	0.234	0.815	0.367
O × F1	**11.396**	**0.001**	**5.359**	**0.021**	**7.018**	**0.008**
O × F2	2.279	0.131	1.727	0.189	0.695	0.405
**Cd × F1**	**5.019**	**0.025**	3.160	0.075	0.001	0.970
Cd × F2	1.422	0.233	1.468	0.226	0.075	0.784
O × E	**4.953**	**0.026**	**4.989**	**0.026**	1.594	0.207
Cd × E	0.973	0.324	0.892	0.345	1.726	0.189
F1 × E	0.179	0.673	0.863	0.353	0.153	0.696
F2 × E	1.512	0.219	0.747	0.388	0.072	0.788
O × Cd × F1	0.048	0.827	0.881	0.348	0.684	0.408
O × Cd × F2	3.340	0.068	0.025	0.875	**6.864**	**0.009**
O × Cd × E	**6.614**	**0.010**	**11.566**	**0.001**	**4.472**	**0.034**
O × F1 × E	2.726	0.099	**5.718**	**0.017**	0.439	0.508
O × F2 × E	0.000	0.994	1.493	0.222	0.084	0.773
Cd × F1 × E	1.316	0.251	1.283	0.257	0.606	0.436
Cd × F2 × E	0.158	0.691	**6.541**	**0.011**	2.925	0.087
O × Cd × F1 × E	0.165	0.684	1.356	0.244	**5.547**	**0.019**
O × Cd × F2 × E	3.495	0.062	**4.988**	**0.026**	0.733	0.392
**Random effects**	SD		SD		SD	
Species identity	0.163		0.108		0.000	
Genus	0.000		0.107		0.024	
Plant number	0.000		0.000		0.004	
Residual	0.343		0.159		0.015	

*Note:* All variables were square‐root transformed. Values were in bold when *p* < 0.05.

### 3.1 Plant Performance of Native and Invasive Species

Total and aboveground mass of invasive plants was significantly promoted under earthworm addition (*χ* ^*2*^ = 4.953, *p* < 0.05 and *χ* ^*2*^ = 4.989, *p* < 0.05 of O × E in Table 1; Figure 1a; Figure S2a). Earthworm presence mitigated the loss of invasive species biomass under Cd contamination, but not in native plants (*χ* ^*2*^ = 6.614, *p* < 0.05 and *χ* ^*2*^ = 11.566, *p* < 0.01 of O × Cd × E in Table 1; Figure 1a; Figure S2a). RMF of plants under Cd and earthworm remained in invasive species but increased in native species (*χ* ^*2*^ = 4.472, *p* < 0.05 of O × Cd × E in Table 1; Figure 1b). In addition, earthworm addition increased the tolerance index of invasive species under Cd contamination, while not in native species (*χ* ^*2*^ = 5.232, *p* < 0.05 of O × E in Table S3; Figure 1c).

### 3.2 Plant Performance of Different Functional Groups

**FIGURE 2:**
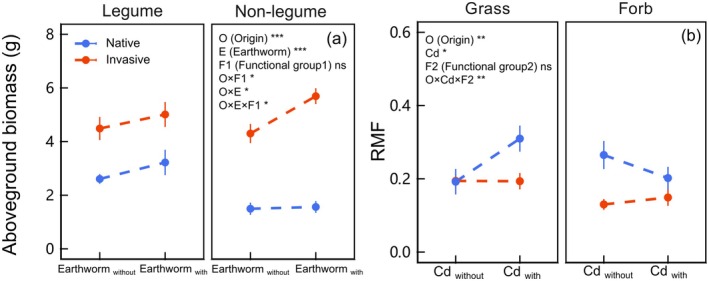
Effects of earthworm addition (with vs. without) on aboveground biomass among different origins (native vs. invasive) and functional group plants (legume vs. nonlegume) (panel a). Effects of Cd contamination (with vs. without) on RMF among different origins and functional group plants (grass vs. forb) (panel b). Values were means ± standard error (SE). *p < 0.05, **p < 0.01, ***p < 0.001, ns *p* > 0.1.

The biomass of invasive species differed significantly from that of native species, particularly among nonlegume (*χ* ^*2*^ = 11.396, *p* < 0.01 and *χ* ^*2*^ = 5.359, *p* < 0.05 of O × F1 in Table 1; Figure S3a; Figure 2a). The biomass of invasive plants was exceeded within the same functional group, except for the belowground biomass of legume (*χ* ^*2*^ = 8.208, *p* < 0.01 of O × F1 in Table S2; Figure S3b). Furthermore, earthworms had no impact on legume biomass while significantly increasing aboveground biomass in invasive nonlegume (*χ* ^*2*^ = 5.718, *p* < 0.05 of O × F1 in Table 1; Figure 2a).

In nonlegume, Cd decreased the belowground biomass of invasive grass, but not in invasive forb (*χ* ^*2*^ = 12.231, *p* < 0.001 of O × Cd × F2 in Table S2; Figure S4a). RMF and R/S were increased in native grass and decreased in native forb under Cd contamination, while they remained in invasive plants (*χ* ^*2*^ = 6.864, *p* < 0.01 and *χ* ^*2*^ = 7.187, *p* < 0.01 of O × Cd × F2 in Table 1, Table S2 and Figure 2b, Figure S4b).

**FIGURE 3:**
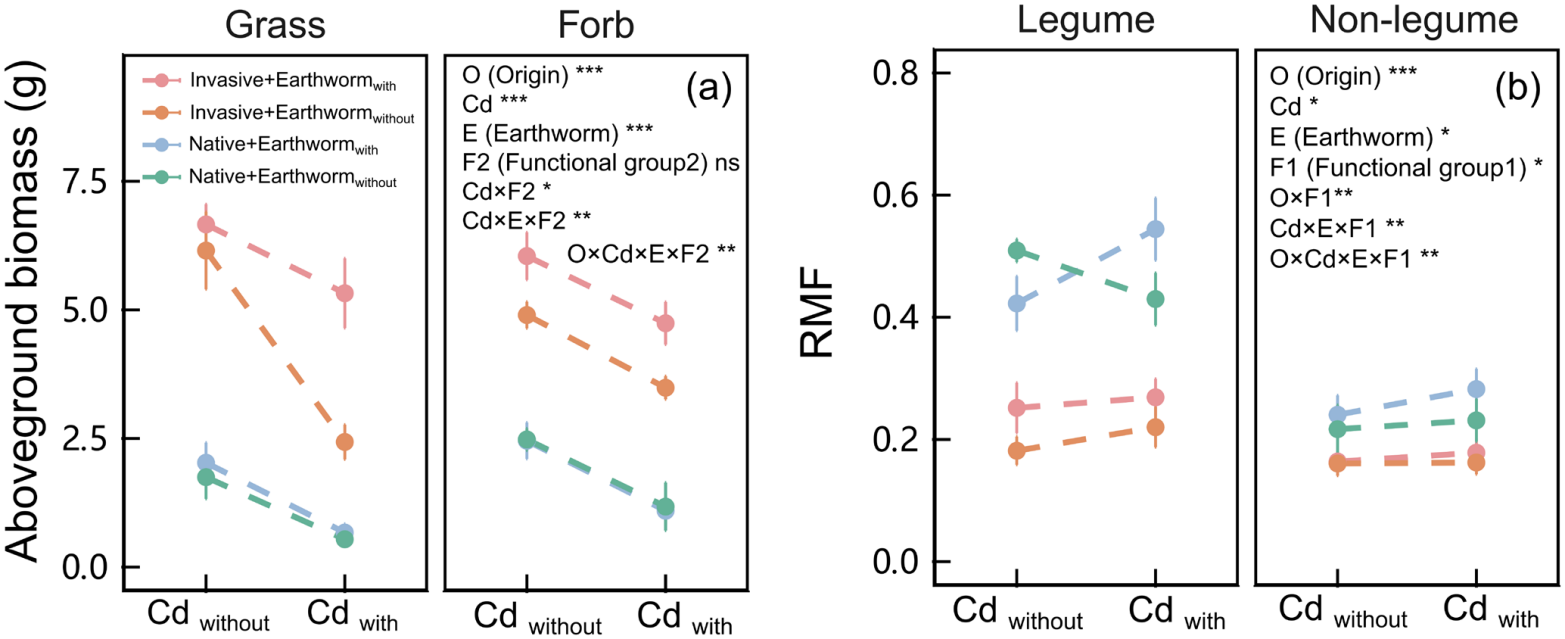
Effects of earthworm addition (with vs. without) and Cd contamination (with vs. without) on aboveground biomass (panel a) and RMF (panel b) among different origins (native vs. invasive) and functional groups of plants (grass vs. forb; legume vs. nonlegume). Values were means ± standard error (SE). *p < 0.05, **p < 0.01, ***p < 0.001, ns *p* > 0.1.

Earthworm mitigated the negative effects of Cd contamination on aboveground biomass in invasive nonlegume, particularly in invasive grass (*χ* ^*2*^ = 4.988, *p* < 0.05 of O × Cd × F2 × E in Table 1; Figure 3a). Similarly, earthworm increased the tolerance index of invasive nonlegume under Cd contamination, while invasive grass increased even more (*χ* ^*2*^ = 6.811, *p* < 0.01 of O × F2 × E in Table S3; Figure S5a). On the other hand, earthworms did not contribute to the biomass of invasive legume under Cd (Figure S6). Furthermore, RMF and R/S ratio were decreased under Cd contamination in native legume, while these trends were reversed in the presence of earthworm (*χ* ^*2*^ = 5.547, *p* < 0.05 and *χ* ^*2*^ = 5.419, *p* < 0.05 of O × Cd × F1 × E in Table 1, Table S2 and Figure 3b, Figure S7).

### 3.3 Lost Mass of Earthworm

**FIGURE 4:**
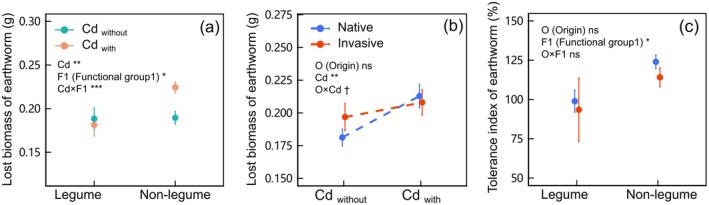
Lost mass of earthworm within different functional groups (legume vs. nonlegume) (panel a) and origin (invasive vs. native) of plants (panel b) under Cd addition (with vs. without). The tolerance index to Cd of earthworms among different functional groups and origins of plants (panel c). Values were means ± standard error (SE). *p < 0.05, **p < 0.01, ***p < 0.001, † 0.1 > *p* > 0.05, ns *p* > 0.1.

Three earthworms escaped, and the remained earthworms were active despite their mean mass decreasing by up to 47.1% at the end of the experiment. The addition of Cd had negative effects on earthworms within nonlegume and led to reducing mass, while not within legume (*χ* ^*2*^ = 14.167, *p* < 0.001 of Cd × F1 in Table 2; Figure 4a). Similarly, earthworm mass remained stable in invasive species under Cd, whereas a significant decline was observed in native species (*χ* ^*2*^ = 3.802, *p* = 0.051 of O × Cd in Table 2; Figure 4b). Furthermore, the tolerance index of earthworms (calculated based on the lost mass, and a higher value represented a lower tolerance to Cd) within nonlegume was higher than that in legume (F = 6.730, *p* < 0.05 of F1 in Table S3; Figure 4c).

**TABLE 2:** Effects of species origin, Cd contamination, plant functional group1, plant functional group2 and their interactions on lost mass of earthworm.

Fixed effects	Lost mass of earthworm	
	*χ* ^*2*^	*p*
Species origin (O)	1.440	0.230
Cd contamination (Cd)	**11.778**	**0.001**
Functional group1 (F1)	**3.877**	**0.049**
Functional group2 (F2)	0.073	0.787
O × Cd	3.802	0.051
O × F1	0.667	0.414
O × F2	0.211	0.646
Cd × F1	**14.167**	**0.000**
Cd × F2	0.119	0.730
O × Cd × F1	0.156	0.693
O × Cd × F2	1.907	0.167
**Random effects**	SD	
Species identity	0.000	
Genus	0.000	
Pot number	0.048	
Residual	0.267	

*Note:* The lost mass of earthworm were log‐transformed. Values were in bold when *p* < 0.05.

### 3.4 Structural Equation Model (SEM)

**FIGURE 5:**
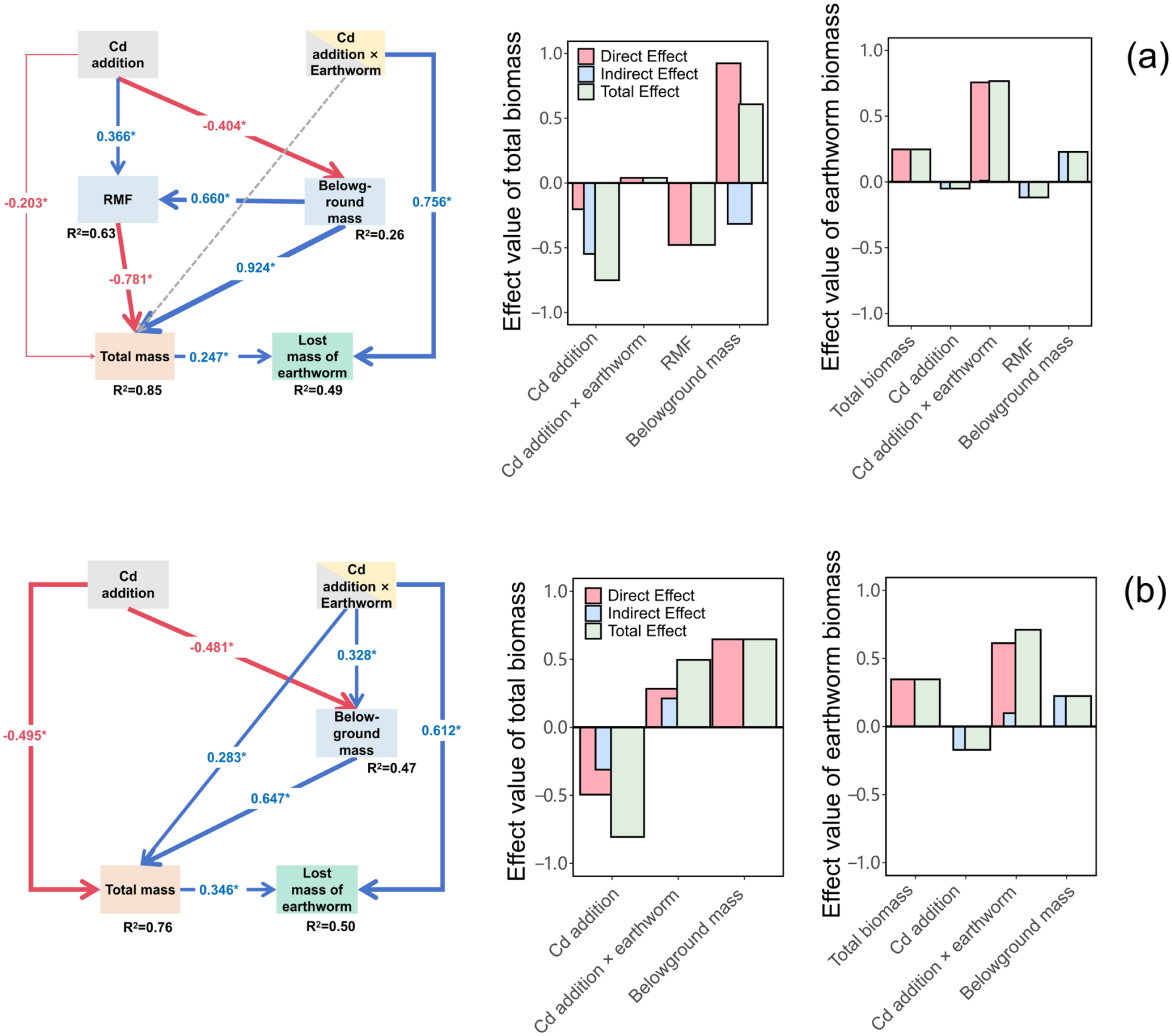
Piecewise structural equation models (pSEMs) for linking among Cd addition, earthworm addition and their interaction for total biomass of plants and lost mass of earthworm in non‐legume (grass and forb). The effect value of direct and indirect were shown in native species (a), and alien invasive species (b). Blue lines represented positive effects, while red lines represented negative effects. Bold lines and marked asterisks indicated statistically significant paths.

SEM explained 85% and 49% of the variability in plant biomass and the loss of earthworm mass in native nonlegume (Figure 5a). Similarly, 76% and 50% of invasive nonlegume (Figure 5b). Cd had negative effects on biomass by altering RMF in native nonlegume (standardized coefficient = 0.366, *p* < 0.05 in Figure 5a), but not in invasive nonlegume. Moreover, earthworm mitigated the negative effects of Cd in invasive nonlegume (standardized coefficient = 0.283, *p* < 0.05 in Figure 5b). Furthermore, the positive effects of total biomass on the loss of earthworm mass were observed in non‐legume (standardized coefficient = 0.247, standardized coefficient = 0.346, *p* < 0.05 in Figure 5a,b), but not in invasive legume (Figure S8b).

## 4 Discussion

We found that Cd had negative impacts on the biomass of plants and earthworms, while earthworms had positive effects on the biomass of plants. Earthworms promoted the accumulation of biomass of invasive plants and reduced the loss of aboveground biomass in invasive plants under Cd (Figure 1). Furthermore, we found that the positive effects of earthworms on invasive plant biomass were observed in invasive nonlegume, regardless of the presence of Cd addition (Figures 2a and 3a). Alternatively, Cd did not reduce the aboveground biomass of invasive legume under earthworm addition (Figure S6). Therefore, our results indicated that earthworms promote the biomass accumulation of invasive nonlegume under Cd contamination and mitigate the loss of biomass in invasive legume caused by Cd contamination. These findings suggested that earthworms enhanced the performance of invasive species under Cd contamination, exacerbating the competitive advantage of the invasive species.

### 4.1 Effects on Plant Performance

The reduction in plant biomass under Cd contamination has been reported in previous studies (van Groenigen et al. 2014; Liang et al. 2023). Cd is highly toxic to plants and inhibits their growth by accumulating in plant tissues (Rizwan et al. 2016). The mitigating effects of earthworms on Cd contamination are perhaps related to improved soil conditions. Feeding, burrowing, and other activities of earthworms in the soil improve root development, regulate microorganisms, promote phosphorus transfer among plants through the hyphal network, and contribute to carbon fixation and nitrogen mineralization through the formation of stable aggregate castings (Tuffen et al. 2002; Blouin et al. 2013). Additionally, the impacts of earthworms on the soil environment, particularly modifications in physicochemical properties, may reduce the mobility and bioavailability of Cd, which could contribute to mitigating the toxic effects of Cd on plants (Sizmur et al. 2011; Wang et al. 2023).

Earthworms significantly enhanced biomass in invasive species, which intensified their relative advantage over native species (Figure 1). Earthworm activities transform soil properties and increase plant‐accessible nutrient levels by promoting organic matter decomposition, nitrogen mineralization, and carbon solidification (Wurst et al. 2004; Zhang et al. 2013). Compared with native species, invasives exhibit greater adaptability to soil environment changes and utilize nutrient pulse variations more efficiently, thereby benefiting more in fluctuating nutrient environments, which aligns with the fluctuating resource hypothesis (Parepa et al. 2013; Wang et al. 2019). Furthermore, earthworms affect plant growth by secreting mucus and depositing abundant microbial communities from their guts into the soil through castings (Chen et al. 2017). Mucus and castings produced by earthworms have been observed to contain bacteria capable of nitrification (Costello and Lamberti 2008; Blouin et al. 2013), which may also be one of the driving factors for the advantage of invasive plants. Furthermore, we found that the increasing biomass in invasive species was observed only in grass and forb, but not in legume (Figure 2a). It is consistent with previous findings that legume aboveground mass remains unaffected by endogeic earthworms (Blouin et al. 2019). The increasing biomass under endogeic earthworms likely results from nitrogen mineralization, whereas nitrogen‐fixing legumes exhibit limited benefits due to their reduced dependence on soil nitrogen availability (van Groenigen et al. 2014).

We observed that invasive species had a high predominance in both belowground and aboveground biomass compared to native species under Cd (Figure 3a; Figures S4, S6). These are consistent with the results of previous studies (Piola and Johnston 2008; Wang et al. 2021). Although the exact mechanism of resistance to HMs in invasive species remains inconclusive, it is generally believed that invasive species possess better strategies, such as enhanced tolerance and avoidance (Li et al. 2022; Rasafi et al. 2022). Our results also confirmed this, as the tolerance index of the invasive species was significantly higher than that of its native species (Figure 1c). Furthermore, they can mitigate the damage caused by HMs through cloning integration (Wang et al. 2016; Dong et al. 2019; Zhao et al. 2023). However, unlike previous studies, we found that although the belowground biomass of invasive grass decreased, invasive species maintain their growth advantage and high tolerance index over native species (Figure 2b; Figures S4a, S5). Studies have demonstrated that certain invasive grasses exhibit both high Cd accumulation and growth dominance (Danh et al. 2009; Rasafi et al. 2022), which may benefit plants, such as enhancing defense (Dai et al. 2020). Unfortunately, the mass of our samples was insufficient to verify this assumption through quantitative analysis of Cd HM assays. On the contrary, the belowground mass, RMF, and R/S ratio of the invasive legume and forb did not decrease (Figure 2b; Figures S3b, S7). This indicates that they maintained a reasonable allocation of resources without altering the division of labor to achieve a balance between nutrition and survival even under cadmium contamination (Wilson 1988; Yan et al. 2013).

More importantly, we found that the presence of earthworms led to a significant increase in the aboveground mass advantage of invasive species over native species under Cd contamination conditions, which was consistent with our hypothesis (Figure 1). Further, earthworms facilitated invasion under Cd contamination by preventing the biomass loss of legumes or increasing the biomass of nonlegumes (Figure 3b), while concurrently improving tolerance to Cd of nonlegumes (Figure S5). Invasive species often possess optimal leaf structures and physiological characteristics that contribute to high adaptability to HMs, which enable them to gain more advantage than native plants under stress by capturing resources more efficiently (Ilyas et al. 2022). On the other hand, the intestinal wall of earthworms, along with the bacteria present within it, such as *Pseudomonas brenneri* , has the ability to absorb and degrade Cd (Wu et al. 2020), which reduces Cd concentration directly. Additionally, earthworm activity can improve the ability of microorganisms to chelate elements, facilitating the complexation of organic substances with Cd^2+^ and reducing the bioavailability of Cd (Hait and Tare 2012). The decrease in Cd concentration and bioavailability could potentially enhance the advantage of invasive species that are more tolerant to Cd (Afzal et al. 2023). Moreover, nitrogen mineralization caused by endogeic earthworms may increase nonlegume biomass potentially, particularly in invasive species exploiting nitrogen pulses (Parepa et al. 2013; van Groenigen et al. 2014). For legumes, although limited effects in nitrogen mineralization, the reduced Cd bioavailability through earthworm activity could potentially alleviate biomass loss. These patterns imply possible divergent mechanisms in earthworm effects on plant functional groups under Cd contamination.

We also found that Cd and earthworm together changed the R/S ratio and RMF of native species, while not in invasive species (Figure 2b; Figures S4, S7). The R/S and RMF are important indicators of plant health under stress, reflecting different allocation of resources and survival strategies (Agathokleous et al. 2019; Barros et al. 2020). Native plants often experience greater stress compared to invasive plants and prioritize survival by sacrificing root growth instead of bud growth (Wilson 1988). In our study, native grasses invested more nutrients into roots and survival, enhancing their resource acquisition ability. This may also be a manifestation of the tolerance strategy. On the contrary, native forbs increased their aboveground production to improve their ability to utilize photosynthetic products. On the other hand, invaders have a competitive advantage as they have larger belowground and aboveground mass, which allows them to absorb available soil resources and use light more efficiently (Grotkopp and Rejmanek 2007). This enabled invasive species to manage Cd stress effectively without altering survival strategies. In addition, earthworms can provide nitrogen pulses that promote accelerated vegetative growth in invasive plants, further enhancing their competitive advantage (Schmidt and Curry 1999; Lopez et al. 2023).

### 4.2 Effects on Earthworm Mass

The lost mass of earthworm was not significant in legume but increased significantly in nonlegume under Cd contamination conditions (Figure 4a). Previous studies have reported positive effects of legume on earthworm mass (van Eekeren et al. 2009; Rodriguez et al. 2020). The roots of legumes contribute to the improvement of soil properties, creating a favorable environment for earthworm activity (Schmidt et al. 2003). On the other hand, the nitrogen fixation ability of legumes provides a beneficial nitrogen source for earthworms (Whalen and Parmelee 2000). This hypothesis was tested by applying inorganic nitrogen fertilizer in the field (Edwards and Lofty 1982; Schmidt et al. 2003). These mechanisms may contribute to partially offsetting the detrimental effects of HMs on earthworms.

Interestingly, invasive species exhibited a negative impact on the loss of earthworm mass (Figure 4b). Earthworms enhance invasive plant biomass while benefiting from this interaction, suggesting a mutualistic relationship between earthworms and invasive species. This is inconsistent with the results of previous studies. Liu et al. (2020) proposed that the allelopathic effects of invasive plants may hinder the physiological activities of earthworms, causing a detrimental effect on them. Studies indicate that invasive plants can influence soil microbial activity and nutrient content through allelopathic effects and spatial expansion, which may serve as a food source for earthworms (Mcleod et al. 2016; Xu et al. 2022). A meta‐analysis indicated that invasive plants boosted microbivore abundance by 89% and detritivore abundance by 119% (Zhang et al. 2019), confirming the positive effects of invasive species on soil animals and showing partial similarity to our results. The symbiotic relationship between earthworms and invasive plants will gradually amplify the competitive edge of invaders over native species, expediting the invasive process and having a detrimental effect on the biodiversity of local ecosystems.

### 4.3 Direct and Indirect Effects on Biomass and Earthworm Mass

We quantified the effects of Cd exposure and earthworm addition on plant and earthworm mass in invasive and native plants, while eliminating irrelevant pathways with poor correlation (Figure 5). Our findings indicated that Cd had a more pronounced negative impact on underground biomass. The absorption and transport of Cd by roots could result in excessive accumulation of Cd, which inhibited growth and triggered the development of different adaptation strategies. Furthermore, the interaction between earthworms and Cd had a negative overall effect on biomass in native plants, while it had a positive effect on biomass in invasive plants. This finding supported the results mentioned above (Figure 3). In all groups, except for invasive legumes, there was a significant association between plant biomass and loss of earthworm mass. This discrepancy might be due to variations in the analytical methods used.

In summary, earthworms promoted the accumulation of invasive plant biomass under Cd contamination, thereby posing a potential threat to native biodiversity and ecosystem stability. Furthermore, our study revealed mutualistic interactions between invasive species and earthworms, highlighting the critical needs for earthworm population management in Cd‐contaminated areas to disrupt their feedback. However, several limitations warrant consideration. The endogeic earthworm *P. guillelmi* selected in this study, which exhibits robust burrowing, nitrogen mineralization, and transport capabilities, likely amplifies the effects on plants compared to other earthworms, particularly enhancing invasive plant growth (Zheng and Li 2009; van Groenigen et al. 2014). Additionally, the lack of quantification of Cd content in plants and earthworms, coupled with the absence of soil nutrient and microbial analyses, prevents understanding of Cd translocation and nutrient cycling in soil–plant–earthworm systems. Furthermore, greenhouse conditions may limit direct extrapolation to natural ecosystems, where environmental factors could synergize with earthworms and Cd contamination to exacerbate invasion processes. Future research should prioritize field experiments integrating multiple environmental stressors, quantify Cd fluxes across soil–plant–earthworm systems, and explore microbial‐mediated mechanisms. Expanding functional groups comparisons of earthworms would also clarify taxon‐specific roles in invasion processes under HM contamination.

## 5 Conclusions

Our results indicated that Cd reduced and earthworm increased plant biomass, varying by plant origin and functional groups. Cd contamination promoted the biomass dominance of invasive species, but decreased the biomass and changed the R/S and RMF of native species. Earthworms consistently enhanced the aboveground biomass of invasive non‐legume species regardless of Cd contamination, while having no effects on native species. On the other hand, earthworms did not change the biomass without Cd but reduced biomass loss of legume under Cd contamination. Thus, earthworm and Cd contamination can facilitate the invasion process of invasive species, especially nonlegume, posing a potential threat to the biodiversity of local ecosystems. These findings highlight earthworm management as a potential strategy to mitigate invasive species spread under Cd contamination and provide a framework for balancing biodiversity conservation and HM remediation in contaminated habitats. Future research should integrate global change factors into field studies to quantify HM transfer and define the role of microorganisms in soil–plant–earthworm systems, refining the mechanisms of plant invasion under HM contamination.

## Author Contributions

**Rui‐Feng Zhang:** conceptualization (equal), investigation (equal), methodology (equal), project administration (equal), writing – original draft (lead), writing – review and editing (lead). **Ai‐Di‐Na Yisilamu:** formal analysis (equal), investigation (equal), methodology (equal), writing – original draft (supporting). **Cong‐Ying Zhao:** formal analysis (equal), investigation (equal), methodology (equal), project administration (equal), writing – original draft (supporting). **Yu‐Jian Guo:** investigation (equal), methodology (equal), writing – original draft (supporting), writing – review and editing (supporting). **Xue Zhang:** investigation (supporting), writing – review and editing (supporting). **Shou‐Shuai Zhang:** investigation (supporting), writing – review and editing (supporting). **Zhao‐Gui Yan:** conceptualization (equal), methodology (equal), writing – review and editing (equal). **Yan‐Feng Bai:** conceptualization (supporting), investigation (supporting), resources (equal), writing – review and editing (equal). **Yong‐Jian Wang:** conceptualization (lead), data curation (equal), funding acquisition (lead), resources (lead), supervision (lead), validation (lead).

## Conflicts of Interest

The authors declare no conflicts of interest.

## Supporting information


Data S1.

## Data Availability

The experimental data and code are given in Supporting Information and Data S1.
