# Supplementary material for: Plant–Earthworm Interaction Favors Invasive Alien Plants Over Natives in Cd‐Contaminated Environments
Source: Ecol Evol. 2025 Jun 30;15(7):e71538. doi: 10.1002/ece3.71538 (PMC12207486; doi:10.1002/ece3.71538)

**Table S1** Information of four target invasive alien and six native plant species used in the experiment.

| Species | Family | Status | Native range | Invasion rank^#^ | Functional groups | Source of species materials | Cultivation methods |
| --- | --- | --- | --- | --- | --- | --- | --- |
| *Alternanthera philoxeroides* (Mart.) Griseb. | Amaranthaceae | Invasive alien | South America | 1 | Forb | Field collection at Zhoushan in Zhejiang | Clonal reproduction |
| *Alternanthera sessilis* (L.) DC | Amaranthaceae | Native | Asia | *-* | Forb | Field collection at Zhoushan in Zhejiang | Clonal reproduction |
| *Sphagneticola trilobata* (L.) Pruski. | Asteraceae | Invasive alien | South America | 2 | Forb | Field collection at Zhaoqing in Guangdong | Clonal reproduction |
| *Sphagneticola calendulacea* (L.) Pruski | Asteraceae | Native | Asia | - | Forb | Field collection at Zhaoqing in Guangdong | Clonal reproduction |
| *Paspalum dilatatum* Poir. | Poaceae | Invasive alien | South America | 3 | Grass | Field collection at Wuhan in Hubei | Clonal reproduction |
| *Paspalum distichum* L. | Poaceae | Native | Asia | - | Grass | Field collection at Wuhan in Hubei | Clonal reproduction |
| *Pennisetum purpureum* Schum | Poaceae | Invasive alien | Africa | 3 | Grass | Seed company in Henan | Sexual reproduction |
| *Pennisetum alopecuroides* (L.) Spreng | Poaceae | Native | Asia | - | Grass | Seed company in Henan | Sexual reproduction |
| *Trifolium pratense* L. | Leguminosae | Invasive alien | Europe, North Africa | 2 | Legume | Field collection at Wuhan in Hubei | Sexual reproduction |
| *Melilotus officinalis* (L.) Pall | Leguminosae | Native | Asia, Europe | - | Legume | Seed company in Henan | Sexual reproduction |
| *Crotalaria pallida* Ait. | Leguminosae | Invasive alien | Africa | 3 | Legume | Seed company in Henan | Sexual reproduction |
| *Melilotus albus* Medik. | Leguminosae | Native | Asia, Europe | - | Legume | Seed company in Henan | Sexual reproduction |

Status and habitat information are based on the Flora of China (www.efloras.org), Scientific Database of China Plant Species (DCP) (http://www.plants.csdb.cn/eflora), Wan et al. (2012) and Ma (2013). ^#^ Invasion rank: 1 – highly invasive alien species in China, i.e. those alien species that cause huge economic and ecological losses, and irreversible changes in natural environments in China; 2 – serious invasive alien species in China, i.e. those alien species that cause major economic and ecological losses, and changes in natural environments in China. The invasion rank information is from Ma (2013).

References:

Ma, J.S., 2013.The checklist of the Chinese invasive plants. Higher Education Press.

Wan, F.H., Liu, Q.Y., Xie, M., et al., 2012. Color illustrations of invasive alien plants in China. Science Press.

**Table S2** Effects of species origin (invasive alien vs. native), Cd contamination (with vs. without), earthworm addition (with vs. without), plant functional group1 (F1: legume vs. non-legume), plant functional group2 (F2: forb vs. grass) and their interactions on Root-shoot ratio (R/S) of plant species. All variables were square-root transformed. Values were in bold when *P* < 0.05.

|  | Belowground biomass | |  | R/S | |
| --- | --- | --- | --- | --- | --- |
| Fixed Effects | *χ*2 | *P* |  | *χ*2 | *P* |
| Species origin (O) | 0.530 | 0.467 |  | **10.776** | **0.001** |
| Cd contamination (Cd) | **30.209** | **0.000** |  | **7.998** | **0.005** |
| Functional group1 (F1) | **4.007** | **0.045** |  | **4.169** | **0.041** |
| Functional group2 (F2) | 0.033 | 0.855 |  | 0.379 | 0.538 |
| Earthworm (E) | **17.504** | **0.000** |  | **4.562** | **0.033** |
| O × Cd | 2.749 | 0.097 |  | 1.144 | 0.285 |
| O × F1 | **8.208** | **0.004** |  | **5.810** | **0.016** |
| O × F2 | **4.849** | **0.028** |  | 0.816 | 0.366 |
| Cd × F1 | 1.555 | 0.212 |  | 0.033 | 0.855 |
| Cd × F2 | 0.063 | 0.801 |  | 0.004 | 0.950 |
| O × E | 1.527 | 0.217 |  | 2.097 | 0.148 |
| Cd × E | 1.398 | 0.237 |  | 1.202 | 0.273 |
| F1 × E | 0.060 | 0.807 |  | 0.120 | 0.729 |
| F2 × E | 0.668 | 0.414 |  | 0.060 | 0.807 |
| O × Cd × F1 | 0.423 | 0.515 |  | 0.485 | 0.486 |
| O × Cd × F2 | **12.231** | **0.000** |  | **7.187** | **0.007** |
| O × Cd × E | 0.404 | 0.525 |  | **4.442** | **0.035** |
| O × F1 × E | 0.335 | 0.563 |  | 0.316 | 0.574 |
| O × F2 × E | 0.028 | 0.867 |  | 0.176 | 0.675 |
| Cd × F1 × E | 0.015 | 0.902 |  | 0.414 | 0.520 |
| Cd × F2 × E | 3.300 | 0.069 |  | 3.559 | 0.059 |
| O × Cd × F1 × E | 0.955 | 0.329 |  | **5.419** | **0.020** |
| O × Cd × F2 × E | 2.416 | 0.120 |  | 0.660 | 0.417 |
| ***Random*** Effects |  | SD |  |  | SD |
| Species identity | 0.057 |  |  | 0.000 |  |
| Genus | 0.135 |  |  | 0.075 |  |
| Plant number | 0.019 |  |  | 0.010 |  |
| Residual | 0.230 |  |  | 0.041 |  |

**Table S3** Effects of species origin (invasive alien vs. native), earthworm addition (with vs. without), plant functional group1 (F1: legume vs. non-legume), plant functional group2 (F2: forb vs. grass) and their interactions on tolerance index of plants or earthworms. All variables were logarithmically transformed. Values were in bold when *P* < 0.05.

|  | Tolerance index of plants | |  | Tolerance index of earthworms | |
| --- | --- | --- | --- | --- | --- |
| Fixed Effects | *χ^2^* | *P* |  | F | *P* |
| Species origin (O) | **4.535** | **0.033** |  | 0.955 | 0.366 |
| Functional group1 (F1) | 3.259 | 0.071 |  | **6.146** | **0.048** |
| Functional group2 (F2) | 0.998 | 0.318 |  | 0.003 | 0.956 |
| Earthworm (E) | 1.362 | 0.243 |  | **-** | **-** |
| O × F1 | 2.533 | 0.111 |  | 0.002 | 0.968 |
| O × F2 | 0.394 | 0.530 |  | 1.118 | 0.331 |
| O × E | **5.232** | **0.022** |  | **-** | **-** |
| F1 × E | 0.920 | 0.337 |  | **-** | **-** |
| F2 × E | 0.002 | 0.962 |  | **-** | **-** |
| O × F1 × E | 0.000 | 0.985 |  | **-** | **-** |
| O × F2 × E | **6.811** | **0.009** |  | **-** | **-** |
| ***Random Effects*** | SD |  |  |  |  |
| Species identity | 0.232 |  |  | - |  |
| Genus | 0.042 |  |  | - |  |
| Residual | 0.158 |  |  | 0.022 |  |

**Fig. S1** Hypothesized direct and indirect effects of earthworm addition, Cd addition and their interaction on plant biomass (total mass, aboveground mass and belowground mass) and the change of earthworm biomass. Black arrowed represented hypothesized positive effects, while red arrowed represent negative effects.


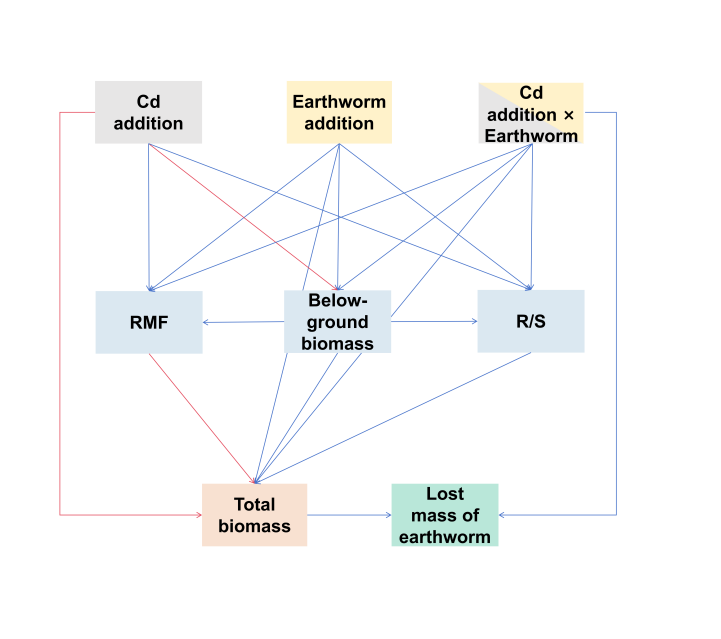


**Fig. S2** Aboveground mass (a) and Root-shoot ratio (b) of all invasive and native plant species under earthworm and Cd addition. Values were means ± standard error (SE).


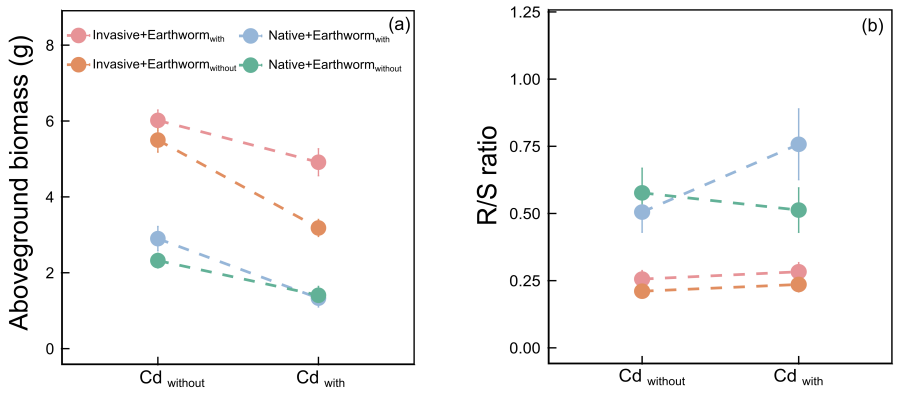


**Fig. S3** Total biomass (a), belowground biomass (b) of all invasive and native plant species in different functional groups (legume and non-legume). Values were means ± standard error (SE).


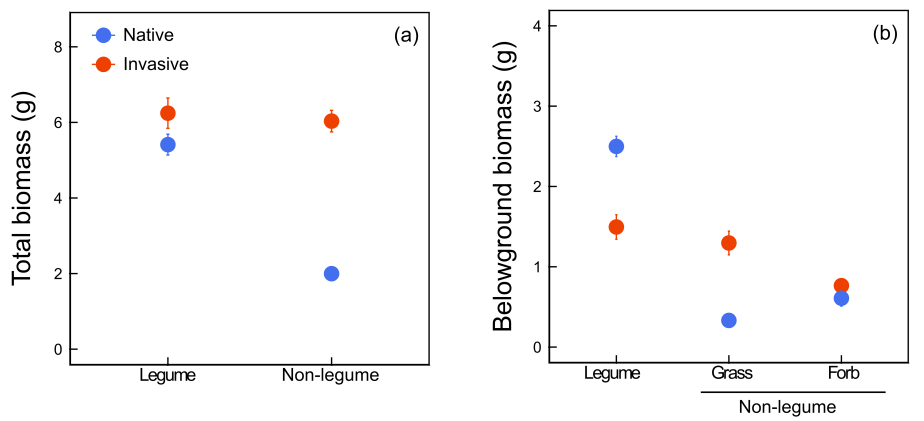


**Fig. S4** Belowground biomass (a) and Root-shoot ratio (b) of native and invasive species in different groups (grass and forb) under Cd addition conditions.


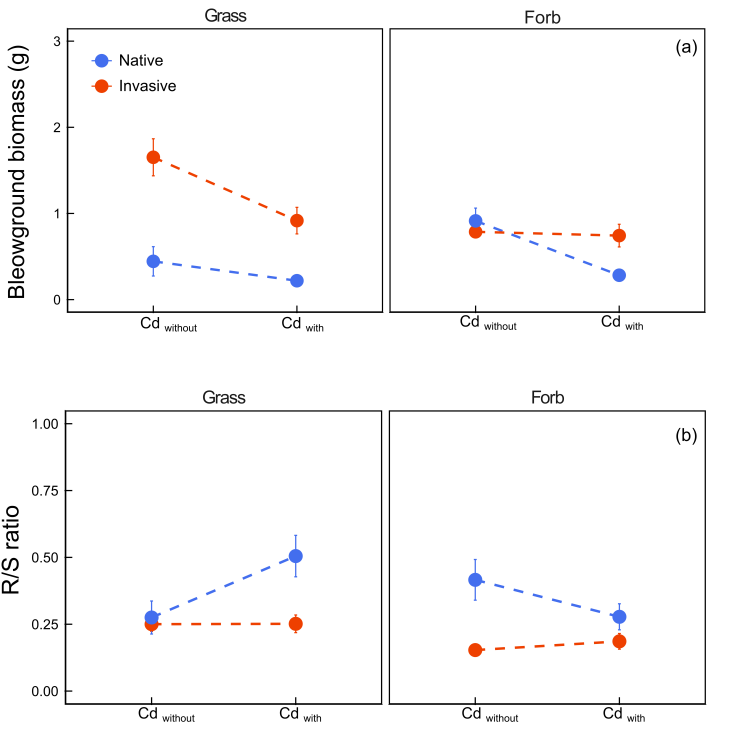


**Fig. S5** Tolerance index of all invasive and native plant species in non-legume (grass and forb) under earthworm. Values were means ± standard error (SE).


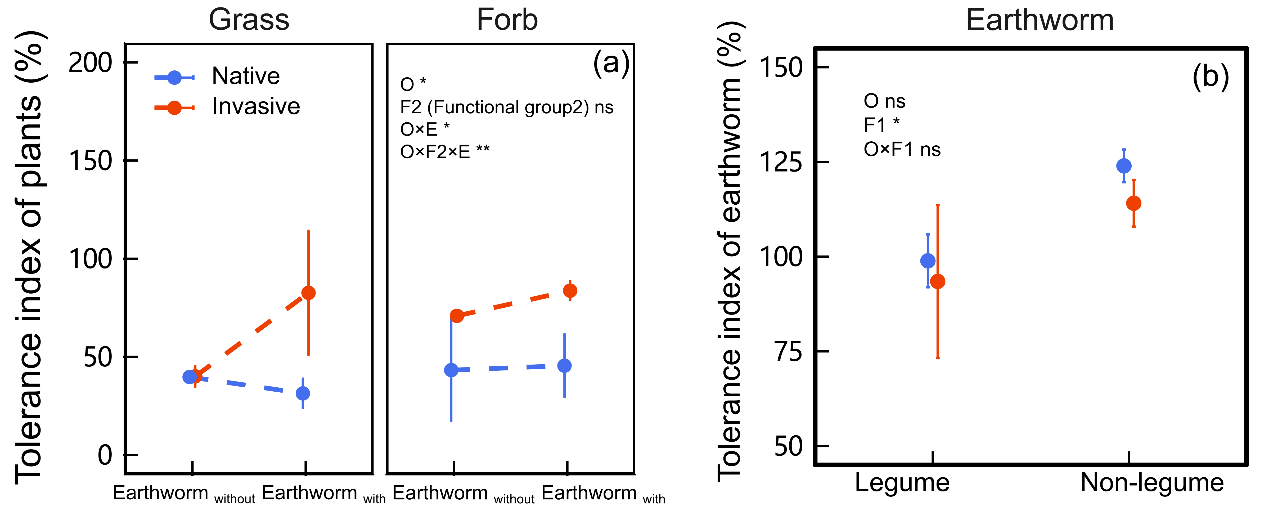


**Fig. S6** Aboveground biomass of all invasive and native plant species in legume and non-legume (grass and forb) under earthworm and Cd addition. Values were means ± standard error (SE).


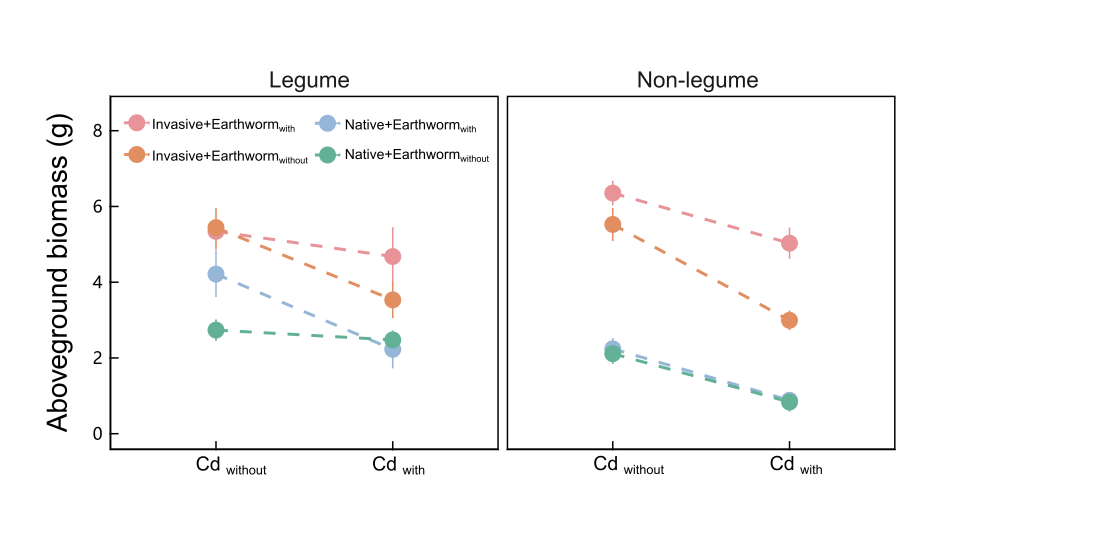


**Fig. S7** Root-shoot ratio of native or invasive different function groups (legume and non-legume) under Cd addition and earthworm addition conditions.


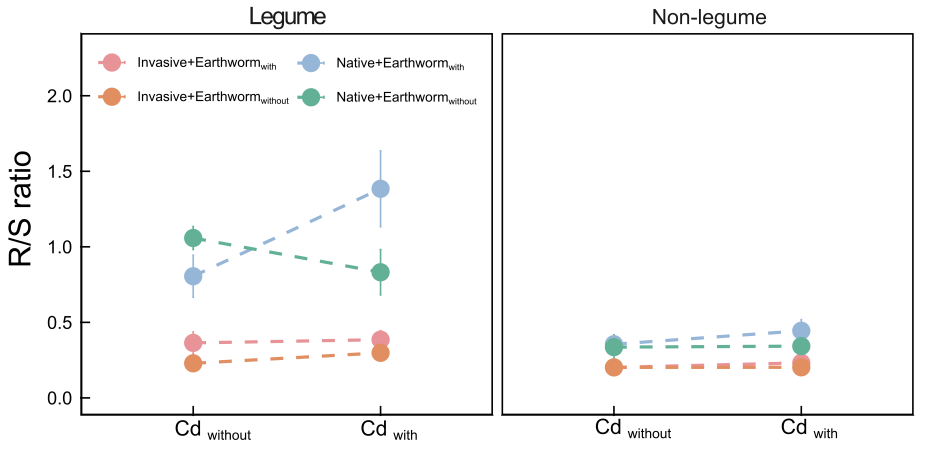


**Fig. S8** Piecewise structural equation models (pSEMs) for linking among Cd addition, earthworm addition and their interaction for total biomass, belowground biomass of plants and lost mass of earthworm in legume. The direct and indirect effects and their effect value in native species (a), and alien invasive species (b). Blue lines represented positive effects, while red lines represented negative effects. Bold lines and marked asterisks indicate statistically significant paths.


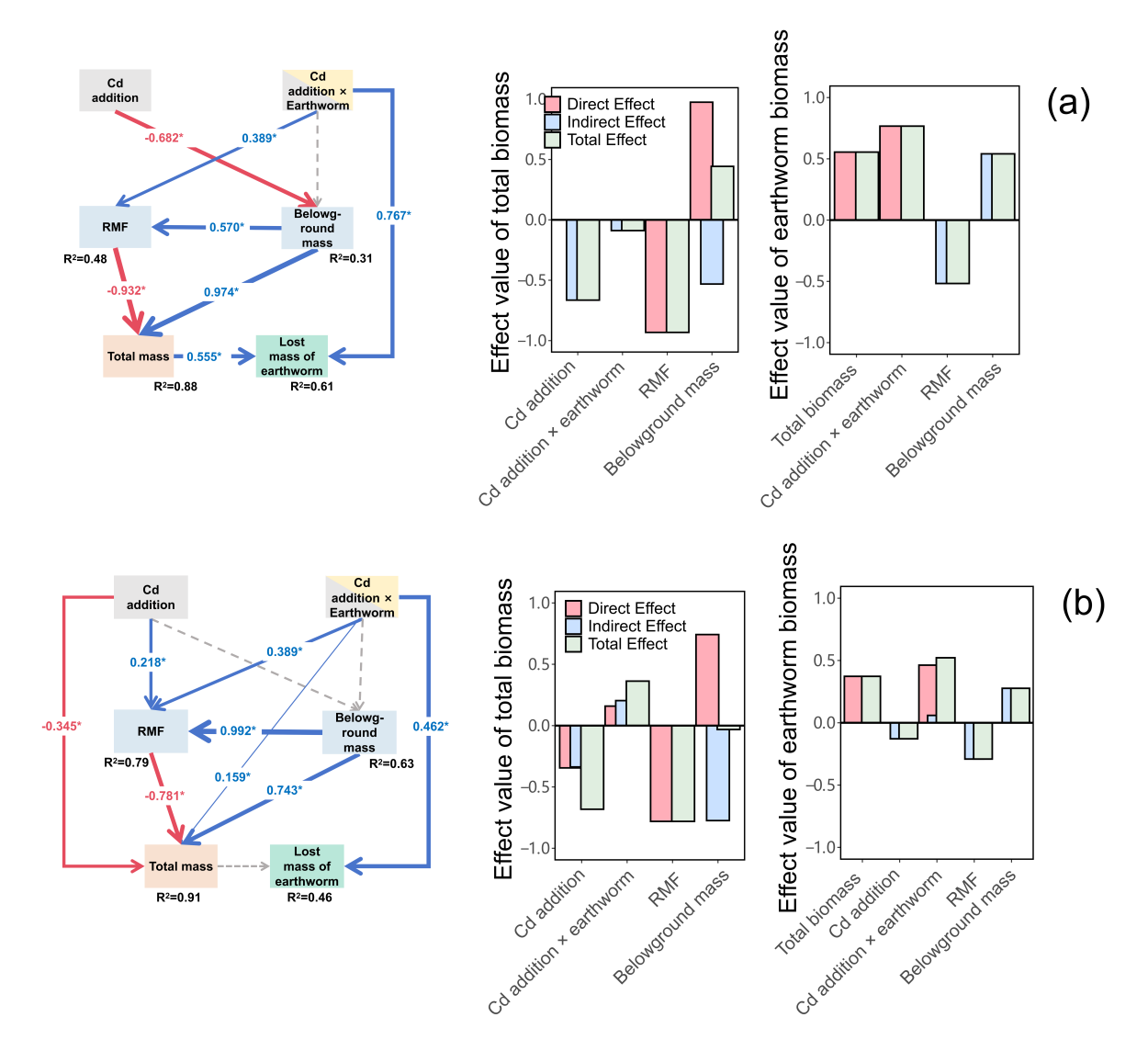

Supplement: Supplementary file 1 — Data S1. [file ECE3-15-e71538-s001.zip › clean_ECE-2025-01-00137_Supporting_Information20250428.docx]
